# A Reference Hand-Book of the Medical Sciences

**Published:** 1885-12

**Authors:** 


**A Reference Hand-Book of the Medical Sciences.** Being a complete and convenient work of reference for information upon topics belonging to the entire range of scientific and practical medicines, and consisting of a series of concise essays and brief paragraphs, arranged in the alphabetical order of the topics of which they treat. Prepared by writers who are experts in their respective departments. Illustrated by chromo-lithographs and fine wood engravings. Edited by ALBERT H. BUCK, M. D. Vol. I. New York: Wm. Wood & Co.

The enterprise of Messrs. Wm. Wood & Co. is only equaled by the ability and completeness with which they carry out their great undertakings. The present volume is the first of a magnificent work of eight volumes of about 800 pages each, the scope of which is fairly outlined in the title above. Dr. Buck is admirably qualified for the editorship of such a work, and in its compilation he has called to his aid many of the most able men in the profession. The present and first volume is, indeed, a splendid one. The topics treated, arranged in alphabetical order, are carried as far as Ca. Matters of a practical nature, the diagnosis and treatment of disease, receive the lion's share of the space; but such departments as medical botany, embryology, physiological and pathological chemistry, military and naval surgery, etc., are fairly represented.

The illustrations are abundant and excellent, and several full-page chromo-lithographs appear. This volume is one of practical worth to the physician, and if the succeeding ones are equal to the standard of this, — and the name of the publishers almost guarantee it, — the complete work will demand a place in every physician's library, and will be a most valuable addition to the long list of splendid works which Messrs. Wood & Co. have issued.

